# Mutants Disrupted in the Type III Secretion System of *Bradyrhizobium elkanii* BLY3-8 Overcame Nodulation Restriction by *Rj*_3_-genotype Soybean

**DOI:** 10.1264/jsme2.ME19151

**Published:** 2020-03-26

**Authors:** Miku Shobudani, Aung Zaw Htwe, Takeo Yamakawa, Matsujiro Ishibashi, Hirohito Tsurumaru

**Affiliations:** 1 Graduate School of Agricultural Science, Kagoshima University, Kagoshima, Japan; 2 Department of Agronomy, Yezin Agricultural University, Yezin, Myanmar; 3 Faculty of Agriculture, Kyushu University, Fukuoka, Japan; 4 Faculty of Agriculture, Kagoshima University, Kagoshima, Japan

**Keywords:** *Bradyrhizobium*, *Rj*_3_, soybean, nodulation, T3SS

## Abstract

*Bradyrhizobium elkanii* BLY3-8 does not form nodules on the roots of *Rj*_3_-genotype soybean (cultivar D-51). This is a cultivar-specific nodulation restriction. The genes *A6X20_40975* and *A6X20_41030* in strain BLY3-8 were predicted to encode the transcriptional activator and apparatus of the type III secretion system (T3SS) (the proteins TtsI and RhcJ), respectively. Mutants disrupted in these genes overcame the nodulation restriction. These results suggest that an effector injected via T3SS into *Rj*_3_-genotype soybean is involved in nodulation restriction by *Rj*_3_-genotype soybean.

*Bradyrhizobium* bacteria form nodules on the roots of soybean plants (*Glycine max*) (Kuykendall, 2015). In these nodules, *Bradyrhizobium* bacteria convert nitrogen into ammonia, which is used for host plant growth. In return, the bacteria receive carbohydrates from soybean for subsistence (Prell and Poole, 2006). However, this symbiotic relationship is sometimes restricted in some *Bradyrhizobium* bacteria strains. For example, the nodulation of *B. japonicum* Is-34 is restricted by *Rj*_4_-genotype soybean (Hayashi *et al.*, 2012). We recently revealed that the *MA20_12780* gene, encoding the putative “effector” protein of the type III secretion system (T3SS) in *B. japonicum* Is-34, is involved in nodulation restriction (Tsurumaru *et al.*, 2015). T3SS is a needle-like apparatus that is used to drive bacterial proteins into host plant cells (Deakin and Broughton, 2009; Staehelin and Krishnan, 2015). T3SS-translocated bacterial proteins are termed “effector” proteins or “nodulation outer proteins (Nops)” (Staehelin and Krishnan, 2015; Cao *et al.*, 2017). Thus, *Rj*_4_-genotype soybean may recognize the putative T3SS effector (MA20_12780 protein) of *B. japonicum* Is-34 as a virulence factor. The *Rj*_4_ gene in soybean was previously reported to encode a thaumatin-like protein (TLP) belonging to pathogenesis-related (PR) protein family 5 (Hayashi *et al.*, 2014; Tang *et al.*, 2014; Tang *et al.*, 2016). Similarly, the nodulation of *B. diazoefficiens* USDA 122 was restricted by *Rj*_2_-genotype soybean (Hayashi *et al.*, 2012). The *Rj*_2_ gene in soybean has been reported to encode a resistance (R) protein against microbial pathogens (Yang *et al.*, 2010; Sugawara *et al.*, 2019). Sugawara *et al.* (2018) recently revealed that NopP in *B. diazoefficiens* USDA 122 was recognized by *Rj*_2_-genotype soybean as a virulence factor. Plant responses such as T3SS effector-induced nodulation restriction are termed effector-triggered immunity (ETI) (Cao *et al.*, 2017).

We previously reported that *B. elkanii* BLY3-8 nodulation was restricted by *Rj*_3_-genotype soybean (cultivar D-51) (Htwe and Yamakawa, 2017). This is cultivar-specific nodulation restriction because the nodulation of *B. elkanii* BLY3-8 was not restricted by non-*Rj*_3_ genotype soybean cultivars (*e.g.* Yezin-3, Yezin-6, Bragg, and Fukuyutaka) (Htwe and Yamakawa, 2017). A *Rj*_3_ gene has not yet been identified in soybean (Hayashi *et al.*, 2012). To verify whether T3SS (effectors) is involved in nodulation restriction by *Rj*_3_-genotype soybean, as in the case of *B. japonicum* Is-34 and *B. diazoefficiens* USDA 122, we disrupted the *ttsI* and *rhcJ* genes in *B. elkanii* BLY3-8 and investigated the nodulation abilities of these mutants in *Rj*_3_-genotype soybean. The *ttsI* and *rhcJ* genes encode transcriptional activators for the T3SS (Krause *et al.*, 2002) and T3SS apparatus (Viprey *et al.*, 1998; de Lyra *et al.*, 2006), respectively (Tsukui *et al.*, 2013). Thus, if T3SS in *B. elkanii* BLY3-8 is involved in nodulation restriction by *Rj*_3_-genotype soybean, the mutants disrupted in the *ttsI* and *rhcJ* genes of *B. elkanii* BLY3-8 may overcome this restriction.

Table S1 lists the bacterial strains and plasmids used in the present study. *B. elkanii* strains were grown at 30°C in modified HM medium (hereafter called HMm medium) by adding 0.1% L-arabinose and 0.03% yeast extract (Cole and Elkan, 1973; Tsurumaru *et al.*, 2015). *Escherichia coli* strains were grown at 37°C in Luria-Bertani (LB) medium (Sambrook *et al.*, 1989).

In *B. elkanii*, the organization of the T3SS gene cluster of strain USDA 61 has been extensively examined (Okazaki *et al.*, 2009). Therefore, to identify the T3SS gene cluster in strain BLY3-8, genes in the T3SS cluster in strain USDA 61 (NCBI accession number FM162234) were used as a query in a BLASTN search (Altschul *et al.*, 1990) against all CDSs in the draft genome of *B. elkanii* BLY3-8 (NCBI accession number LWUI00000000) (Htwe *et al.*, 2016). The disruption of the *ttsI* and *rhcJ* genes in the T3SS gene cluster in strain BLY3-8 was achieved using a single-crossover recombination strategy (Okazaki *et al.*, 2009). Mutants disrupted in these genes and wild-type strain BLY3-8 were inoculated into *Rj*_3_-genotype soybean (cultivar D-51). The methods used to identify the T3SS gene cluster, disrupt the *ttsI* and *rhcJ* genes, and plant assays are described in the Supplementary Materials and Methods.

A T3SS gene cluster was identified on contig 61 of the draft genome of *B. elkanii* BLY3-8 (NCBI accession number NZ_LWUI01000058.1) (Fig. 1a). Among the 49 CDSs in the T3SS gene cluster of strain USDA 61, 34 were detected in the cluster in strain BLY3-8 by a BLASTN analysis. These genes had >79% sequence identity to those in strain BLY3-8. The genes *ttsI* and *rhcJ* in strain USDA 61 had 92 and 89% identities to the *A6X20_40975* and *A6X20_41030* genes, respectively, in strain BLY3-8. Six *tts* boxes were detected in the T3SS gene cluster of strain BLY3-8 (Fig. 1b).

The genes *A6X20_40975* and *A6X20_41030* encoding the TtsI and RhcJ proteins in strain BLY3-8 (Fig. 1a) were disrupted by the single-crossover recombination method, resulting in the mutants MttsI and MrhcJ, respectively. Experimental results confirming the single-crossover recombination event in the mutants MttsI and MrhcJ are shown in Fig. S1. Wild-type strain BLY3-8, the mutant MttsI, and mutant MrhcJ were inoculated into *Rj*_3_-genotype soybean (cultivar D-51). The wild-type strain induced leaf chlorosis in soybean, and plants were smaller than those inoculated with mutants (Fig. 2a). This may have been due to a nitrogen deficiency because of nodulation restriction (Uchida, 2000). Strain BLY3-8 formed no effective nodules (Fig. 2b), but a few ineffective nodules (Fig. 2a), which is consistent with our previous findings (Htwe and Yamakawa, 2017). In contrast, the mutants MttsI and MrhcJ both overcame this nodulation restriction (Fig. 2b); they formed red sections on the root nodules (Fig. 2a), indicating that they were effective nodules (Gwata *et al.*, 2003). No significant differences were observed in effective nodule numbers between the two mutants MttsI and MrhcJ (Tukey’s test, *n*=4, *P*>0.05) (Fig. 2b).

*B. elkanii* BLY3-8 did not form effective nodules on the roots of *Rj*_3_-genotype soybean. However, mutants disrupted in the *ttsI* and *rhcJ* genes in *B. elkanii* BLY3-8 overcame this nodulation restriction (Fig. 2). The present results clearly demonstrated that the bradyrhizobial T3SS apparatus was involved in nodulation restriction by *Rj*_3_-genotype soybean. Therefore, ETI in *Rj*_3_-genotype soybean may cause nodulation restriction, and a T3SS effector protein in strain BLY3-8 appears to directly or indirectly interact with a resistance (R) protein or pathogenesis-related (PR) protein in *Rj*_3_-genotype soybean. As described above, neither the causal gene in strain BLY3-8 not an *Rj*_3_ gene in soybean have been identified.

## Citation

Shobudani, M., Htwe, A. Z., Yamakawa, T., Ishibashi, M., and Tsurumaru, H. (2020) Mutants Disrupted in the Type III Secretion System of *Bradyrhizobium elkanii* BLY3-8 Overcame Nodulation Restriction by *Rj*_3_-genotype Soybean. *Microbes Environ ***35**: ME19151.

https://doi.org/10.1264/jsme2.ME19151

## Supplementary Material

Supplementary Material

## Figures and Tables

**Fig. 1.**
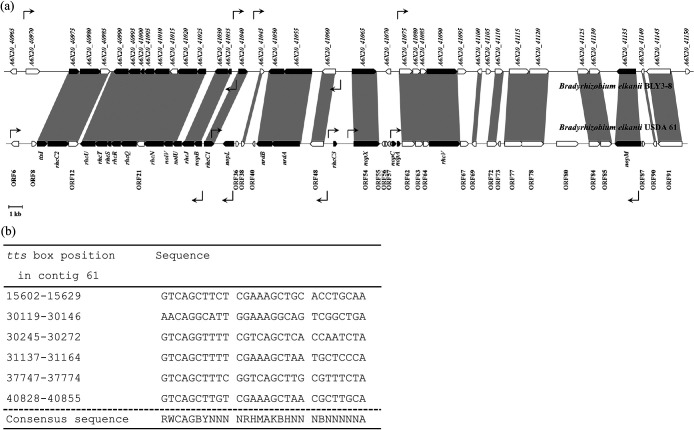
Features of the T3SS gene cluster in *B. elkanii* BLY3-8. (a) Genetic organization of the T3SS gene cluster in strain BLY3-8 and its comparison to that in strain USDA 61. The predicted gene orientations and sizes are indicated by an arrow box; the black arrow box indicates the genes associated with T3SS, and the white arrow box indicates putative genes. The locations and directions of the *tts* box sequence are shown by rectangular arrows with solid lines. (b) Multiple sequence alignment of the putative *tts* boxes in strain BLY3-8.

**Fig. 2.**
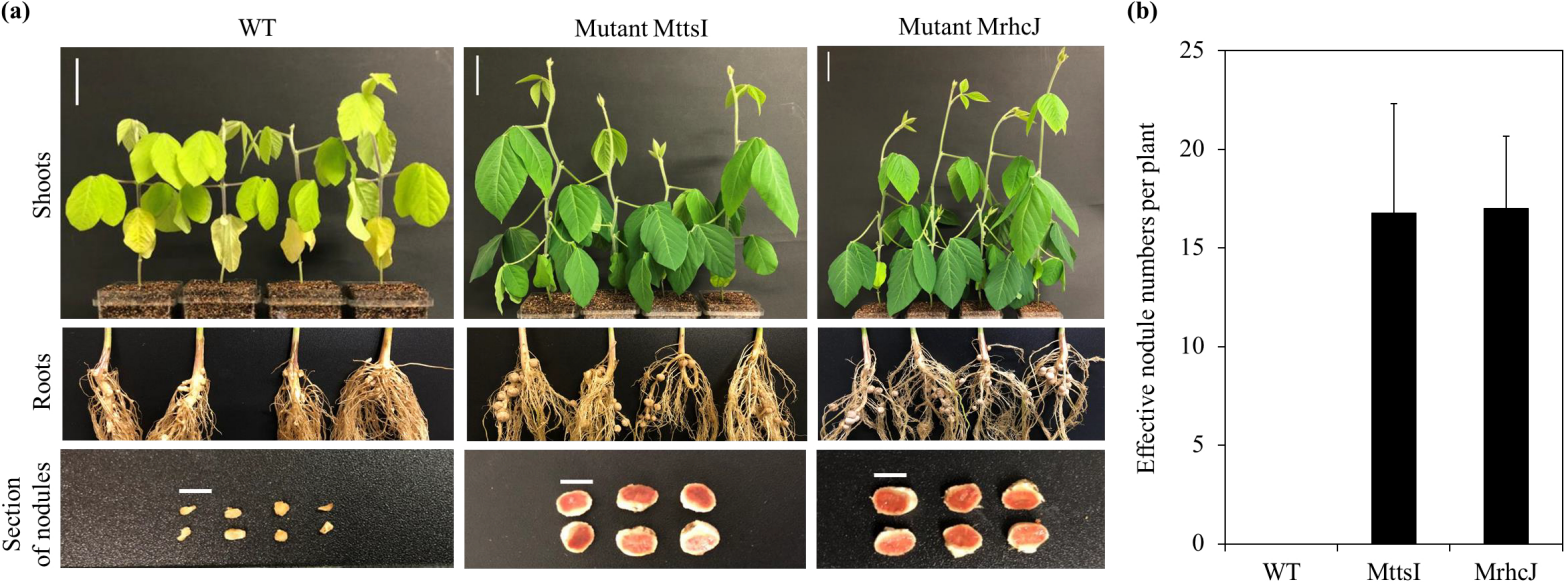
Nodulation phenotype of *Rj*_3_-genotype soybean (cultivar D-51) inoculated with the wild-type strain (WT), MttsI mutant, and MrhcJ mutant. (a) Shoots, roots, and a section of nodules of *Rj*_3_-genotype soybean inoculated with the indicated strains. The white scale bars of shoots and the section of nodules indicate 5‍ ‍cm and 5‍ ‍mm, respectively. (b) Effective nodule numbers on roots of *Rj*_3_-genotype soybean (cultivar D-51) inoculated with the indicated strains (*n*=4). After four weeks of cultivation, nodule numbers were counted. Error bars indicate standard deviations.
