# Supplementary material for: Mutants Disrupted in the Type III Secretion System of Bradyrhizobium elkanii BLY3-8 Overcame Nodulation Restriction by Rj3-genotype Soybean
Source: Microbes Environ. 2020 Mar 26;35(2):ME19151. doi: 10.1264/jsme2.ME19151 (PMC7308571; doi:10.1264/jsme2.ME19151)
Supplement: Supplementary file 1 — Supplementary Material [file 35_19151_s1.pdf]

Supplementary materials

Title: Mutants disrupted in the type III secretion system of *Bradyrhizobium elkanii* BLY3-8 overcame nodulation restriction by *Rj*$_3$-genotype soybean.

Authors: Miku Shobudani, Aung Zaw Htwe, Takeo Yamakawa, Matsujiro Ishibashi, and Hirohito Tsurumaru

Corresponding author: Hirohito Tsurumaru, Faculty of Agriculture, Kagoshima University,
1-21-24 Korimoto, Kagoshima 890-0065, Japan.
E-mail: k1759347@kadai.jp ; Tel. and Fax: +81-99-285-8635.

Supplementary Materials and Methods

Identification of T3SS gene cluster in *B. elkanii* BLY3-8

Genes in the T3SS cluster in strain USDA 61 (NCBI accession number FM162234) were analyzed by a BLASTN search using the NCBI BLAST+ program (Camacho *et al*., 2009) against all CDSs in the draft genome of *B. elkanii* BLY3-8 (NCBI accession number LWUI00000000). A map for the T3SS gene cluster was drawn using drawGeneArrows3 software (http://www.ige.tohoku.ac.jp/joho/labhome/tool.html) (Fig. 1a).

The binding site of TtsI is known to be located in the upstream region of the T3SS apparatus and effector genes, termed the *tts* box (Krause *et al*., 2002; Zehner *et al*., 2008). The putative *tts* box in the T3SS gene cluster of strain BLY3-8 was identified using the DNA Pattern Find program (http://www.bioinformatics.org/sms2/dna_pattern.html) with the consensus sequence of the *tts* box (5′-RWCAGBYNNNNRHMAKBHNNNBNNNNNA-3′) (Tsurumaru *et al*., 2015; Zehner *et al*., 2008). Multiple sequence alignment analyses of putative *tts* boxes found in strain BLY3-8 was performed with the ClustalW program in BioEdit software, version 7.2.5 (http://www.mbio.ncsu.edu/bioedit/bioedit.html) (Hall, 1999).

Disruption of *ttsI* and *rhcJ* genes in *B. elkanii* BLY3-8

A single-crossover recombination strategy (Okazaki *et al*., 2009) was used to disrupt *ttsI* and *rhcJ* genes in strain BLY3-8. The internal region of the *ttsI* gene in strain BLY3-8 was amplified by PCR with paired primers: F-ttsI (5′-AAATCTAGACTACCACATTCTTTTGCTCG-3′) and R-ttsI (5′-AAATCTAGACAAATGTTCGAGAATGCACA-3′) (the *Xba* I site is underlined). Genomic DNA of strain BLY3-8 was purified using an ISOPLANT kit (Nippon Gene Co., Ltd., Tokyo, Japan). The PCR mixture (50 µL) contained 0.25 µL of Ex Taq HS DNA polymerase

(TaKaRa Bio Inc., Shiga, Japan), 5 μL of 10 × buffer (Ex Taq buffer; TaKaRa Bio Inc.), 4 μL of dNTP mixture (2.5 mM each) (TaKaRa Bio Inc.), 1 μL of each primer (50 μM), genomic DNA solution, and sterilized water (up to the final volume). The cycling conditions were as follows: 98°C for 1 min, 30 cycles of 98°C for 10 s, 52°C for 30 s, and 72°C for 30 s. The PCR products were purified using the AMPure XP (Beckman Coulter, Inc., Tokyo, Japan) according to the manufacturer's protocol. The purified PCR product was digested with *Xba* I (Toyobo Co., Ltd., Osaka, Japan). Plasmid pK18mob (Schäfer *et al*., 1994) was digested with *Xba* I and then treated with bacterial alkaline phosphatase (BAP) (Nippon Gene Co., Ltd.). The *Xba* I- digested PCR product was ligated into the *Xba* I site of a pK18mob plasmid with T4 DNA Ligase (Nippon Gene Co., Ltd.), resulting in a pttsI plasmid. This plasmid was transformed into *E. coli* JM109 (Toyobo Co., Ltd.). *Xba* I digestion, BAP treatment, ligation, and transformation were all performed according to the manufacturers' protocols. Plasmid pttsI was mobilized from *E. coli* JM109 into *B. elkanii* BLY3-8 through triparental mating with the helper plasmid pRK2013 (Figurski and Helinski, 1979). The plasmid pttsI-harboring *E. coli* JM109 and plasmid pRK2013-harboring *E. coli* HB101 were cultivated using LB medium containing kanamycin (Km) at 50 μg mL^{-1}. Briefly, 1 mL culture of *E. coli* JM109 (plasmid pttsI), 1 mL culture of *E. coli* HB101 (plasmid pRK2013), and 3 mL culture of *B. elkanii* BLY3-8 were harvested by centrifugation (21,500 g, 5 min, 10°C). The cell pellets were washed twice with HMm medium. The cell pellet of strain BLY3-8 was suspended into a 1 mL HMm medium. The suspension was then mixed with cell pellets of *E. coli* JM109 (plasmid pttsI) and *E. coli* HB101 (plasmid pRK2013). This mixture was harvested by centrifugation (21,500 g, 5 min, 10°C) and then re-suspended into 100 μL of HMm medium. The suspended culture was spread onto a cellulose acetate membrane filter (Advantec, Tokyo, Japan; pore size 0.45 μm). Mating was performed on HMm plates at 30°C for two days. After mating, cells were suspended in 1 mL of HMm medium, and serial 10-fold dilutions of the

suspension were performed (from 10^{-1} to 10^{-9}) using HMm medium. Subsequently, 100 µL aliquots of the diluted cultures were spread onto HMm plates containing 150 µg mL^{-1} Km and 50 µg mL^{-1} polymyxin B. Mutant MttsI was obtained by further single colony isolation using HMm plates containing 50 µg mL^{-1} Km. Disruption of the *ttsI* gene in mutant MttsI was confirmed by PCR with primer sets F-M13/F-MttsI and F-MttsI/R-MttsIs. The primer sequences were as follows: F-M13, 5′-GTTTTCCCAGTCACGACGTT-3′ (http://nippongene.com/siyaku/product/pcr/m13-primer/m13-primer-mix.html [in Japanese; accessed May 2019]); F-MttsI, 5′-GAAGCACTTCCCGATTGCG-3′; and R-MttsI, 5′-CCAACGAGTTCAAGGGTGT-3′. The PCR condition was the same as above, except that the annealing temperature was 53°C. The PCR products were confirmed using 1% agarose gel electrophoresis.

According to the above procedure, the *rhcJ* gene in strain BLY3-8 was also disrupted, resulting in mutant MrhcJ. The paired primers, F-rhcJ (5′-AAATCTAGACATATTCGATTGACCTGCTC-3′) and R-rhcJ (5′-AAATCTAGAACAGATAAGATGCAATGACGA-3′), were used to amplify the *rhcJ* gene's internal region (the *Xba* I site is underlined). The plasmid constructed for this mutation was called prhcJ (Table S1). Two primer sets R-M13/F-MrhcJ and F-MrhcJ/R-MrhcJ were usued for to confirm *rhcJ* gene disruption in mutant MrhcJ. The primer sequences were as follows: R-M13, 5′-CAGGAAACAGCTATGAC-3′ (https://www.promega.jp/products/cloning-and-dna-markers/molecular-biology-enzymes-and-reagents/puc_m13-sequencing-primers/?catNum=Q5401 [in Japanese; accessed May 2019]); F-MrhcJ, 5′-TCTCTACAGCAAGATTCAGG-3′;, and R-MrhcJ, 5′-GCATCGGAGGTGATCTTTT-3′.

Plant assays

Mutants MttsI and MrhcJ were cultivated using 20 mL of HMm medium containing 50

μg mL^{-1} Km. After cultivation, cell pellets were harvested by centrifugation (21,500 g, 5 min, 10°C). The cell pellets were washed twice with HMm medium and finally re-suspended into 30 mL of sterilized water. These suspensions were used as the inoculant for the soybean. The inoculant solution of strain BLY3-8 was also prepared, using the above procedure, except that Km was not added to HMm medium, and it was used as the control.

Soybean cultivar D-51 was identified as the Rj_3-genotype soybean (Yamakawa et al., 1999; Htwe and Yamakawa, 2017) and was found to be incompatible with *B. elkanii* BLY3-8 (Htwe and Yamakawa, 2017). The seeds' surfaces were sterilized in 1% sodium hypochlorite for 5 min and washed thrice with sterilized water. After immersion in sterilized water for 5 min, the seeds were washed with sterilized water two times. The seeds were placed on sterilized filter paper moistned with sterilized water in a sterilized petri dish, and then germinated for two days at 28˚C. The seedlings were transplanted into a Leonard jar pot (three seedlings per pot) (Tsurumaru *et al*., 2015). At transplantation, inoculation of strain BLY3-8 or its mutants were performed (3 mL of the inoculants per pot). The plants were grown in a plant growth chamber (CHL-301; TOMY SEIKO Co. Ltd., Tokyo, Japan) at 28°C/25˚C under a 16-h light/8-h dark cycle. After five days of cultivation, the plants were thinned to one seedling per pot; after four weeks, nodule numbers were counted.

Supplementary table

Table S1. Bacterial strains and plasmids used in this study

Strains or plasmids	Characteristic(s) [a]	Reference or source
Strains		
Bradyrhizoibum elkanii		
BLY3-8	Wild-type strain	Htwe and Yamakawa, 2017
MttsI	*ttsI* gene-disrupted mutant of BLY3-8, Km^r	This study
MrhcJ	*rhcJ* gene-disrupted mutant of BLY3-8, Km^r	This study
Escherichia coli		
JM109	Host for plasmids prhcJ, pttsI, and pK18mob.	Toyobo Co., Ltd.
HB101	Host for plasmid pRK2013.	Figurski and Helinski, 1979
Plasmids		
prhcJ	pK18mob derivative containg an internal region of *rhcJ* gene, Km^r	This study
pttsI	pK18mob derivative containg an internal region of *ttsI* gene, Km^r	This study
pK18mob	Cloning vector, Km^r	Schäfer *et al.*, 1994
pRK2013	Helper plasmid for mating	Figurski and Helinski, 1979

[a] Km^r, kanamycin resistant.

Supplementary figures

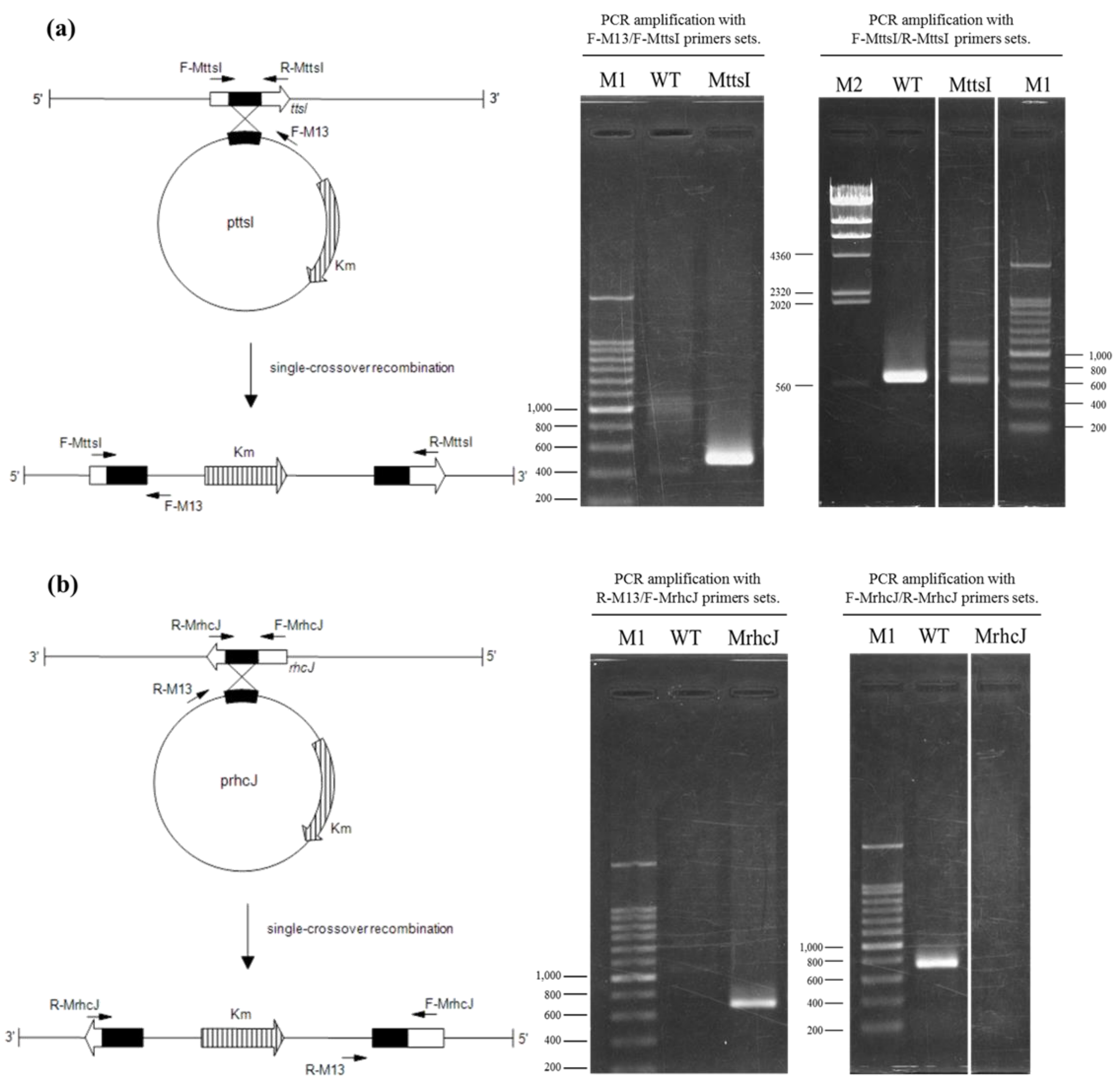

Fig. S1. Confirmation experiment for the single-crossover recombination event. (a) Schematic representation of *ttsI* gene-disruption, and PCR amplification with F-M13/F-MttsI and F-MttsI/R-MttsI primers sets. The expected size of PCR product in mutant MttsI with F-M13/F-MttsI primers set is 485 bp. The expected size of PCR product in strain BLY3-8 (WT) with F-MttsI/R-MttsI primers set is 620 bp. (b) Schematic representation of *rhcJ* gene-disruption, and PCR amplification with R-M13/F-MrhcJ and F-MrhcJ/R-MrhcJ primers sets. The expected size of PCR product in mutant MrhcJ with R-M13/F-MrhcJ

primers set is 694 bp. The expected size of PCR product in strain BLY3-8 (WT) with F-MrhcJ/R-MrhcJ primers set is 750 bp. The PCR products were analyzed on a 1% agarose gel. Lane M1, M2 and WT indicate a 200 bp ladder marker (Toyobo Co., Ltd.), λ/Hind III marker (Nippon Gene, Toyama, Japan) and wild-type PCR product, respectively. Molecular sizes (in bases) are indicated on the left or right.

References

Camacho, C., Coulouris, G., Avagyan, V., Ma, N., Papadopoulos, J., Bealer, K., and Madden, T.L. (2009) BLAST+: architecture and applications. *BMC Bioinf* **10**: 421.

Figurski, D.H., and Helinski, D.R. (1979) Replication of an origin-containing derivative of plasmid RK2 dependent on a plasmid function provided in trans. *Proc. Natl. Acad. Sci. U.S.A* **76**: 1648-1652.

Hall, TA. 1999. BioEdit: a user-friendly biological sequence alignment editor and analysis program for Windows 95/98/NT. *Nucleic Acids Symp. Ser* **41**: 95–98.

Htwe, A.Z., and Yamakawa, T. (2017) Incompatible nodulation of *Bradyrhizobium elkanii* strains BLY3-8 and BLY6-1 with Rj_3 gene-harboring soybean cultivars. *Am. J. Plant Sci* **8**: 178.

Krause, A., Doerfel, A., and Göttfert, M. (2002) Mutational and transcriptional analysis of the type III secretion system of *Bradyrhizobium japonicum*. *Mol. Plant-Microbe Interact* **15**: 1228-1235.

Okazaki, S., Zehner, S., Hempel, J., Lang, K., and Göttfert, M. (2009) Genetic organization and functional analysis of the type III secretion system of *Bradyrhizobium elkanii*. *FEMS Microbiol. Lett*. **295**: 88-95.

Schäfer, A., Tauch, A., Jäger, W., Kalinowski, J., Thierbach, G., and Pühler, A. (1994) Small mobilizable multi-purpose cloning vectors derived from the *Escherichia coli* plasmids pK18

and pK19: selection of defined deletions in the chromosome of *Corynebacterium glutamicum*. *Gene* **145**: 69-73.

Tsurumaru, H., Hashimoto, S., Okizaki, K., Kanesaki, Y., Yoshikawa, H., and Yamakawa, T. (2015) A putative T3SS effector encoded by the *MA20_12780* gene in *Bradyrhizobium japonicum* Is-34 causes the incompatibility with Rj_4 genotype soybeans. *Appl. Environ. Microbiol* **81**: 5812–5819.

Yamakawa, T., Eriguchi, M., Hussain, A.A., and Ishizuka, J. (1999) Soybean preference for *Bradyrhizobium japonicum* for nodulation: nodulation by $Rj_2Rj_3Rj_4$-genotypes isolated from the progenies of a cross between soybean cvs. IAC-2 (Rj_2Rj_3) and Hill (Rj_4). *Soil Sci. Plant Nutr* **45**: 461-469.

Zehner, S., Schober, G., Wenzel, M., Lang, K., and Göttfert, M. (2008) Expression of the *Bradyrhizobium japonicum* type III secretion system in legume nodules and analysis of the associated *tts* box promoter. *Mol. Plant-Microbe Interact* **21**: 1087-1093.
